# Effects of acetylated distarch phosphate on the physicochemical characteristics and stability of the oyster sauce system

**DOI:** 10.3389/fnut.2024.1412314

**Published:** 2024-08-08

**Authors:** Xiao Li, Feixue Zou, Xuemin Kang, Wei Gao, Bo Cui, Jie Sui

**Affiliations:** ^1^State Key Laboratory of Biobased Material and Green Papermaking, Qilu University of Technology, Shandong Academy of Sciences, Jinan, China; ^2^School of Food Science and Engineering, Qilu University of Technology, Shandong Academy of Sciences, Jinan, Shandong, China; ^3^Shandong Academy of Agricultural Science, Jinan, Shandong, China

**Keywords:** acetylated distarch phosphate, oyster sauce, physicochemical properties, interaction, microstructure, stability of food

## Abstract

In this study, the effect of different acetylated distarch phosphate (ADSP) ratios (0, 1%, 2%, 3%, 4%, 5%) on the physicochemical characteristics and stability of the oyster sauce (OS) system was investigated. The texture, water state, interactions, rheological properties, microstructure, and stability of OS samples were analyzed through the texture analyser, low-field nuclear magnetic resonance (LF-NMR), particle diameter and zeta potential, fourier-transform infrared spectroscopy (FTIR), rheometer, and microscopes. The results revealed that the addition of ADSP improved the firmness, consistency, cohesiveness, and water-holding capacity of OS. Moreover, ADSP reduced the average particle size and zeta potential of OS, indicating that electrostatic and steric stabilization existed in the ADSP-OS system. The addition of ADSP enhanced the hydrogen bonding and decreased water mobility for OS system, processing a more continuous and smooth structure. All ADSP-OS samples were typical non-Newtonian fluids with shear-thinning characteristics. In addition, the non-significant instability index changes of ADSP-OS over the whole storage period confirmed the excellent long-term stabilization capability of OS prepared with ADSP. This study provides a theoretical basis for starch-based sauce products and contributes to the development of sauce products.

## 1 Introduction

An oyster is a major maricultural shellfish with tender meat and abundant nutritional values (1). Owing to the higher myofibrillar protein contents (31.10 g/100 g), oysters have earned a nickname “undersea milk” (2). One of the most popular products processed from oysters is known as oyster sauce (OS). This product is frequently consumed with a variety of foods to enhance their natural flavors and improve both their appearance and texture. OS is particularly popular among consumers in the southern provinces of China (such as Guangdong, Fujian, and Hong Kong) and Southeast Asia (3). With the rapid development of convenience and prepared food industries, there is an increasing market demand for sauce products, such as OS.

Generally, the raw components used in preparing OS include oyster concentrate (OC), water, sugar, salt, hydrocolloids, or other flavoring ingredients (3). When used in pre-cooked dishes, OS must withstand the high-temperature heating, low pH, and high shear mixing stages of the manufacturing process and remain stable during the transportation, storage, and heating stages before consumption. However, similar to other sauce products, OS often suffers from undesirable issues such as serum separation, syneresis, or flocculation, which have a negative impact on product quality and consumer acceptance (4). Therefore, manufacturers must add hydrocolloids (such as xanthan gum, locust bean gum, and modified starch) to sauces to provide sufficient viscosity and to stabilize the suspensions for prolonged shelf life (5). Hydrocolloids have been studied in various sauces, including ketchup, white sauce, and chili sauce (6–10). However, studies investigating the potential of hydrocolloids for texture stabilization and improved storage properties in the development of OS are lacking. Furthermore, the quality of sauces was improved with the addition of hydrocolloids, while fundamental problems such as water exudation and texture losses still urgently needed to be resolved.

Modified starch is a popular additive. It is widely used as a texture stabilizer and water retention agent in the preparation of various foods (11–13). Many studies have reported that acetylated starch could improve the storage stability of food products, and it is recommended for the production of salad dressings, canned foods, and puddings (14, 15). Researchers reported that the storage stability of food starch formulations can be improved by acetyl substituent, whilst phosphate cross-links enhance the resistance of systems containing swollen granules against high temperature and shear treatment (16). At the same time, phosphate groups can also enhance the transparency and viscosity of food, which are necessary qualities for sauce products. Acetylated distarch phosphate (ADSP) is commonly used to extend the shelf life of foods (15). However, limited studies have focused on the effect of ADSP on the physicochemical properties and the mode of action of OS. In the present study, ADSP was used in the OS system. The impact of ADSP on the physicochemical characteristics and stability of the OS system was investigated. This research is intended to add value to the development of sauce products.

## 2 Materials and methods

### 2.1 Materials

The OS (carbohydrates: 24 g/100 g, protein: 7.82 g/100 g, fat content: 0) used in the study was purchased from Shajing Co., Ltd. (Shenzhen, China). ADSP (acetyl substitution content: 1.71 g/100 g) was provided by Foshan Summit Starch Technology Co., Ltd. (Guangdong, China). It was modified from waxy corn starch. Salt and white granulated sugar were purchased from the market. Fluorescein isothiocyanate (FITC) with 95% purity was provided by Yuanye Biotechnology Co., Ltd. (Shanghai, China). Absolute ethanol (≥99.7%) was obtained from Tianjin Fuyu Fine Chemical Co., Ltd. (Tianjin, China).

### 2.2 Sample preparation

First, the OS was heated (40% w/w) at 60°C and mixed with different concentrations of ADSP [0%, 1%, 2%, 3%, 4%, and 5% (w/w)], followed by the addition of sugar and salt. Afterward, the mixture was heated to 100°C and held for 10 min, with continuous stirring to obtain a homogeneous system. Finally, six samples were obtained and named as 0starch-OS, 1% ADSP-OS, 2% ADSP-OS, 3% ADSP-OS, 4% ADSP-OS, and 5% ADSP-OS. The prepared OS samples were cooled at 25°C and stored in a refrigerator for further analysis.

### 2.3 Textural analysis

The texture properties of OS samples were evaluated using a texture analyzer (EZ-SX, Shimadzu, Japan). The samples were poured into a circular cylinder (diameter: 50 mm and height: 70 mm), and a back extrusion test was performed using a probe with a diameter of 40 mm. Briefly, when the force of the probe squeeze samples reached 0.05 N, the downward test distance was calculated. Subsequently, the sample was pressed down to a depth of 20 mm at a constant speed of 30 mm/min and then returned to its original position at a speed of 40 mm/min. Four textural parameters were obtained from the tests, including firmness (N) (maximum positive force obtained during compression), consistency (mJ) (positive area of the curve), cohesiveness (N) (maximum negative force in reduction), and viscosity index (mJ) (the area under the curve). Each sample was tested at least five times. The texture of OS was determined at different storage times (0, 10, 20, 30, and 60 days) to reflect its storage stability.

### 2.4 Water-holding capacity

The water-holding capacity (WHC) was assessed using a modified method described by Lan et al. (17). Subsequently, a weighted 40-g OS sample was placed in 50-ml centrifuge tubes. After centrifuging at 3,000 r/min for 15 min, the supernatant was removed, and the mass of the solid part was weighed. The *WHC* was calculated using Equation 1.


(1) $$\begin{array}{l} WHC\left(\%\right)\;=\;\left(M_{2}-M\right)/M_{1}\times 100, \end{array}$$

where M is the weight of the centrifuge tube, M_1_ is the weight of the OS sample (40 g), and M_2_ is the weight of the OS after centrifugation.

### 2.5 Low-field nuclear magnetic resonance (LF-NMR)

The water distribution of OS with or without ADSP was measured using a low-field pulsed NMR analyzer (Newmark Technology, Jiangsu, China). An oil sample was used for calibration before measurement, and then, the samples of the same mass were placed in an NMR tube with a diameter of 25 mm and measured using the Carl-Purcell-Merbo-Gill sequence. During the measurement, the temperature was maintained at 32°C. The typical test parameters were as follows: the echo time was 0.3 ms, the waiting time for repeat sampling was 15,000 ms, the number of slices was 8, and the echo number was 3,000. Each measurement was performed in triplicate.

### 2.6 Particle diameter and zeta potential

The average particle size and zeta potential of samples were measured by dynamic light scattering using a Zetasizer Nano-ZS90 (Malvern Instruments, Worcestershire, UK) analyzer. Specifically, the samples were diluted 100 times with deionized water, and approximately 3 ml of the solution was transferred into the matching cuvette and equilibrated for 120 s before testing.

### 2.7 Fourier-transform infrared spectroscopy (FTIR)

First, the OS samples were freeze-dried. Then, they were ground and sieved through a 100-mesh sieve. The FTIR spectra of the OS samples were measured using a Fourier-transform infrared spectrometer (Nicolet IS10, USA) equipped with a universal ATR attachment. The scanning range was from 4,000 to 400 cm^−1^ with 32 scan times and 4 cm^−1^ resolution. The spectrum was acquired in the range of 1,200–800 cm^−1^ and deconvolved using OMNIC software.

### 2.8 Rheology

The rheological properties of different OS samples were measured using a rotational MCR 302 rheometer (Anton Paar, Graz, Austria). Approximately 10 ml of the OS sample was placed on a parallel plate (50 mm in diameter and 1 mm in gap) and subjected to a shear rate of 0.01–100 s^−1^ to obtain the apparent viscosity. Further fitting analysis was conducted using the power law model (Equation 2).


(2) $$\begin{array}{l} \sigma =K\gamma^{n}, \end{array}$$

where σ is the shear stress (Pa), *K* is the consistency index (Pa·sn), γ corresponds to the shear rate (s^−1^), and n corresponds to the flow behavior index.

The viscoelasticity of ADSP-OS was investigated in the linear viscoelastic region. The elastic modulus (G′), viscous modulus (G″), and the ratio of G″ and G′ (tan δ) were identified in the angular frequency range of 0.1–100 rad/s. Moreover, the strain and gap of this study were 1.0% and 1 mm, respectively.

### 2.9 Fluorescence microscope

The fluorescence microscope (BX53F, Olympus, Japan) was used to observe the morphological distribution of ADSP in the OS system. The OS sample stained with FITC (0.1%, w/v) was placed on a clean glass slide and covered with a coverslip to be observed. The excitation and emission wavelengths of FITC are 488 and 530 nm, respectively.

### 2.10 Atomic force microscope (AFM)

The surface roughness of the OS system was examined using an atomic force microscope (AFM) (Bruker Multimode8, Madison, USA) with a scan size of 5 μm × 5 μm. Approximately 5 μL of the sample that was diluted 800 times with distilled water was dropped onto a cleaved mica surface and air-dried before testing. NanoScope Analysis software was used to collect images, where the bright areas corresponded to peak features in height.

### 2.11 Stability measurement

The stabilization behaviors of the OS stabilized by ADSP were determined using a centrifugal LUMiFuge (L.U.M., Germany). The method is based on accelerated sedimentation and flocculation using centrifugation to provide information on the stability of the sample (18). Approximately 2.0 ml of the sample was collected using needles and placed into a rectangular polycarbonate cuvette. The detection light wavelength was set at 865 nm, the rotation speed was 4,000 rpm, the test temperature was 25°C, and the total separation time was 1.5 h. As the separation time increased, the transmission profile and instability index of the OS were recorded.

### 2.12 Statistical analyses

Each set of experiments was replicated a minimum of three times, and the data are presented as the average value ± standard deviation (SD). ANOVA was performed using SPSS 23, and a *p* < 0.05 was considered statistically significant.

## 3 Results and discussion

### 3.1 Texture properties of OS

**Figure 1:**
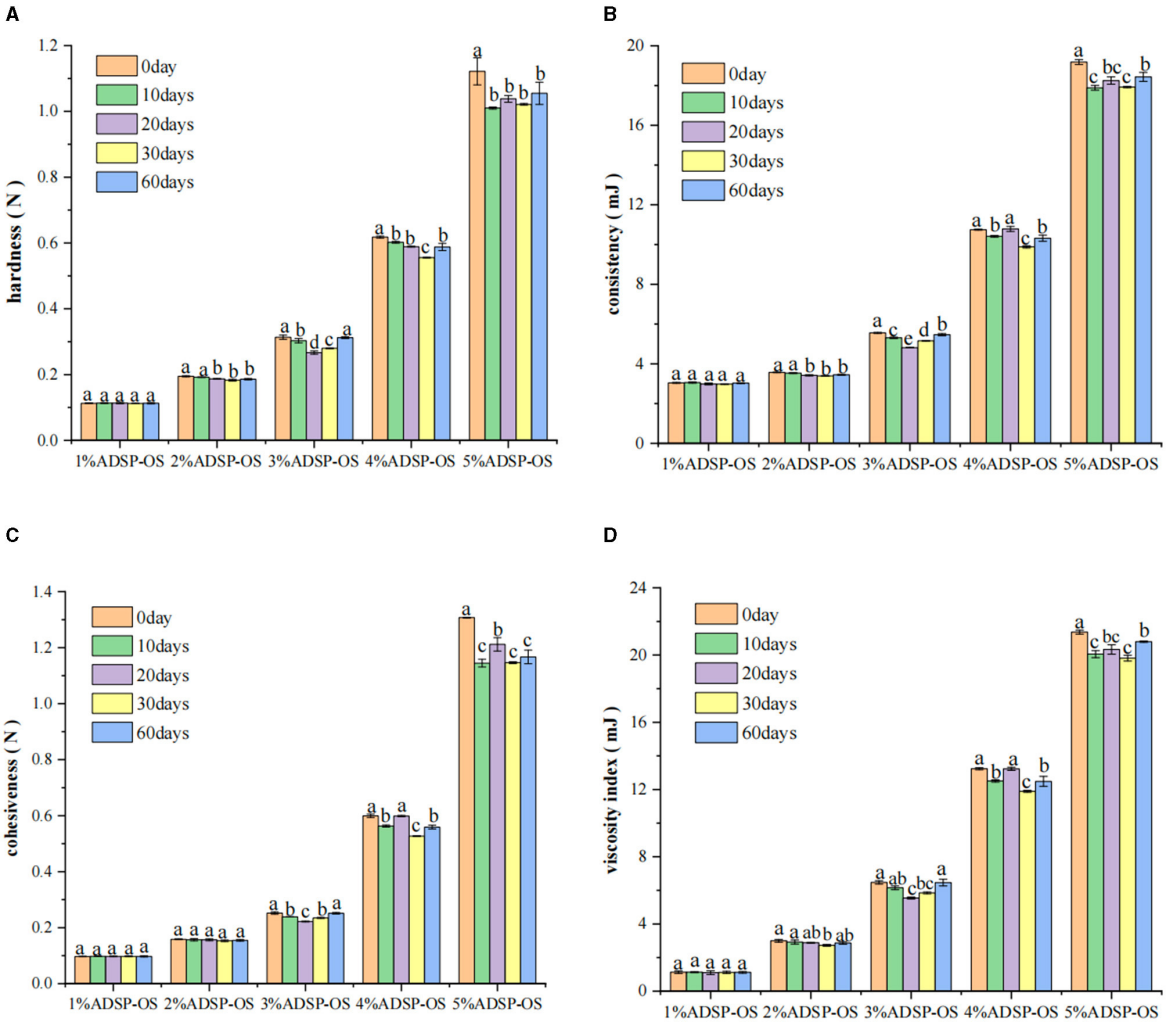
Effects of ADSP addition on the OS system, firmness **(A)**, consistency **(B)**, cohesiveness **(C)**, and viscosity index **(D)**. Different letters indicate significant differences (*P* < 0.05).

Texture is one of the essential reference indicators for evaluating the quality of sauce products. To maintain data accuracy, the use of 0strach-OS was deemed unsuitable for texture determination due to its liquid state. The textural properties of OS samples with different ADSP dosages and stored times are shown in Figure 1. Notably, four texture parameters (firmness, consistency, cohesiveness, and viscosity index) significantly increased in a concentration-dependent manner (*P* < 0.05). The increase in firmness and consistency could be attributed to the swelling and water absorption of starch during the OS preparation. The expanded starch granules filled the sauce system, restricting the movement of other sauce components. Similar research has also found that the starch molecules function as “fillers” in milk gels (19). The authors attributed this effect to the increased viscosity of the sample due to starch molecules binding and orienting water, which consequently dampened the effect of applied stress. In addition, a higher ADSP dosage might promote the formation of a denser structure, leading to an increase in the texture parameters of the OS (firmness: 0.11–1.12 N, consistency: 3.04–19.18 mJ).

It was further found that cold storage changed the texture properties of the OS (Figure 1). As the storage time increased, four texture parameters changed slightly in 1% and 2% ADSP-OS, resulting in a more liquid consistency. The firmness and consistency of 3%, 4%, and 5% ADSP-OS decreased followed by an increase. This change might be attributed to the rearrangement of starch and its reabsorption of water. Overall, there were not many changes in the texture of the OS over the whole storage period. The texture parameters of 5% ADSP-OS were significantly higher than those of other OS, indicating a more rigid system. Notably, 4% ADSP-OS had the approximate firmness, consistency, and cohesiveness values after 10 and 90 days of storage. These results reflected that ADSP enhanced the stability of OS, and the optimal ADSP content for the OS system was found to be 4% (w/w).

### 3.2 WHC of OS

The release of water from the starch-based sauce during storage is known as syneresis. This phenomenon has a detrimental effect on the overall quality of the sauce (7). In this study, we used syneresis to measure the WHC of OS samples, as shown in Table 1. Since OS was processed through boiling, the proteins may have been denatured or resulted in the exposure of hydrophobic groups, and the limited protein content was not sufficient to form a gel structure to retain water (20, 21). Thus, the WHC of 0starch-OS was lower (11.74%). For ADSP-OS, the WHC significantly increased from 46.74% to 97.04% with the increasing ADSP content (1%−5%) (*P* > 0.05). Previous studies have shown that introducing acetyl groups into starch molecules by acetylation leads to repulsion between starch molecules due to steric hindrance, allowing more water molecules to be absorbed (22, 23). Generally, gelatinized starch has water bonding capacity and could form a weak network, which reduces syneresis (24). Moreover, previous research has reported that higher starch concentrations could enhance the interaction between starch and starch, or starch and protein, leading to a higher WHC (25). Consequently, a higher WHC was achieved in the ADSP-OS. In summary, ADSP played a thickening and stabilizing role in the OS system due to its better swelling and water retention properties. In the presence of a limited number of water molecules, high concentrations of starch could increase the competitive adsorption of water, resulting in increased firmness and consistency of the OS, which is consistent with the results of textural properties. We performed the low-field nuclear magnetic resonance (LF-NMR) test to further investigate the OS's water distribution.

**Table 1:** Effect of ADSP on water holding capacity (WHC), LF-NMR relaxation time, and the area percentage of the OS system.

**Sample**	**WHC (%)**	**Relaxation time (ms)**		**Area percentage (%)**	
		**T_21_**	**T_22_**	**A_21_**	**A_22_**
0starch-OS	11.74 ± 0.42^f^	7.84 ± 0.12^a^	141.57 ± 4.58^a^	1.32 ± 0.03^f^	98.68 ± 0.03^a^
1%ADSP-OS	46.74 ± 0.35^e^	2.30 ± 0.08^d^	114.95 ± 3.72^b^	1.54 ± 0.03^e^	98.46 ± 0.03^b^
2%ADSP-OS	60.33 ± 0.60^d^	2.64 ± 0.17^cd^	107.24 ± 3.46^bc^	1.65 ± 0.02^d^	98.35 ± 0.02^c^
3%ADSP-OS	69.75 ± 0.44^c^	2.91 ± 0.19^c^	100.05 ± 3.24^cd^	1.99 ± 0.05^c^	98.01 ± 0.05^d^
4%ADSP-OS	84.36 ± 0.45^b^	3.04 ± 0.19^c^	97.77 ± 3.24^d^	2.36^b^ ± 0.04	97.64 ± 0.04^e^
5%ADSP-OS	97.04 ± 0.24^a^	4.11 ± 0.28^b^	89.07 ± 2.37^e^	2.92 ± 0.04^a^	97.08 ± 0.04^f^

Values are means of triplicate determination ± standard deviation. Means with different letters in the same column are significantly different (P < 0.05).

### 3.3 Water state of OS

Relaxation time (T_2_) can be monitored using LF-NMR to analyze the water distribution and migration in samples without altering the sample structure (26). The present study observed two relaxation components in the OS system, represented as T_21_ and T_22_. According to the previous report with slight modifications: T_21_, with a relaxation time of 0–20 ms, indicated a less mobile water fraction; the peak ranges from 100 to 300 ms corresponded to a more mobile water fraction (15). The T_2_ distributions, including the peak values (T_21_, T_22_) and the area percentages (A_21_, A_22_) associated with the peaks of the OS system, are shown in Table 1. The results revealed that T_2_ values of the OS were significantly reduced (*P* < 0.05) after the addition of ADSP. Specifically, T_22_ of the OS decreased from 141.57 to 89.07 ms, and A_21_ increased from 1.32% to 2.92% when the additive content of ADSP increased to 5%, which suggested that ADSP has a significant effect on water retention in the OS system. This effect might be due to the enhanced interaction between excess ADSP and OS systems, which promotes the formation of compact structures, resulting in a decrease in water mobility. Furthermore, ADSP, with its excellent water absorption and thickening properties, could impede the migration of water molecules. However, the lower-content starch was not effective in filling, leading to higher water mobility and limited water retention. As the amount of ADSP added increased, the water absorption of the OS increased, and more water was converted to tightly bound water. In addition, the starch became closer, which further restricted water mobility. These results were in line with the increased WHC.

### 3.4 Particle diameter and zeta potential

To reveal the interaction between ADSP and the OS system, the particle size and zeta potential of the OS with different dosages of ADSP were investigated and presented in Table 2. The average particle size of the OS was approximately 2,096 nm and that of 0starch-OS was ~3,123 nm, which was larger than that of any other ADSP-OS systems. This phenomenon implied that the highest degree of aggregates formed in the 0starch-OS system. Additionally, it was clear that the average particle size of the OS system progressively decreased from 1,885.46 to 957.33 nm with the addition of ADSP (1%−5%). It was similar to the report on adding hydroxypropyl distarch phosphate to yogurt (27). The ADSP with a smaller particle size played a dispersing role, which might reduce the formation of protein aggregates in the OS system. Meanwhile, the superior adhesive properties of ADSP provided viscosity for the OS, which limited the engagement of proteins. According to Stokes' law, a smaller size of particles is beneficial for maintaining physical stability (28). The changes in the polydispersity index were consistent with the results related to particle size.

**Table 2:** Average particle size, polydispersity index, and zeta-potential of OS, ADSP, and OS with or without ADSP.

**Sample**	**Average particle size (nm)**	**Polydispersity index**	**Zeta-potential**	**1,047/1,022**
OC	2,095.67 ± 58.45^b^	0.76 ± 0.02^b^	−14.57 ± 0.24^f^	–
0starch-OS	3,122.67 ± 87.88^a^	0.87 ± 0.03^a^	−14.97 ± 0.45^f^	–
1%ADSP-OS	1,885.46 ± 42.91^c^	0.84 ± 0.03^a^	−13.87 ± 0.29^e^	0.799 ± 0.002^d^
2%ADSP-OS	1,689.33 ± 30.73^d^	0.79± 0.02^b^	−13.38 ± 0.17^de^	0.823 ± 0.005^d^
3%ADSP-OS	1,506.35 ± 9.29^e^	0.78 ± 0.03^b^	−12.83 ± 0.26^d^	0.868 ± 0.004^c^
4%ADSP-OS	1,328.00 ± 21.65^f^	0.67 ± 0.02^c^	−10.24 ± 0.46^c^	0.886 ± 0.006^c^
5%ADSP-OS	957.33 ± 14.88^g^	0.65 ± 0.03^c^	−9.04 ± 0.30^b^	0.923 ± 0.024^b^
ADSP	290.60 ± 5.72^h^	0.45 ± 0.02^d^	−6.62 ± 0.07^a^	1.097 ± 0.024^a^

Mean values with different letters are significantly different (P < 0.05).

In this section, the zeta potential was also investigated to further explore the stabilization mechanism of ADSP-OS. All samples exhibited a negative charge (Table 2). The zeta potentials of OS, 0starch-OS, and ADSP were −14.57, −14.97, and −6.62 mV, respectively. The two biopolymers with the same type of net charge exhibited electrostatic interaction through surface-selective patch binding. However, the addition of ADSP decreased the OS zeta potential, and the absolute zeta potential of ADSP-OS became less negative as the ADSP content increased. This change might be primarily due to the ADSP with a lower charge covering the charged groups of protein in the OS, thereby influencing the zeta potential value of the system (29). According to the DLVO classical theory, the lower zeta potential values could cause the system particles to flocculate due to the smaller repulsive effect. However, the stability of the OS system was enhanced after adding ADSP. ADSP is a fence-type long-chain molecule that carried negative charges, and has sufficient conformational freedom to avoid aggregation. Thus, introducing bulky acetyl groups in starch could provide a steric hindrance, which helps in stabilizing the OS system (23). It was known that steric hindrance and electrostatic interaction were the two important forces for stabilizing yogurt, emulsions, or other gel systems (30, 31). Therefore, although the zeta potential decreased, the ADSP-added OS exhibited better stability. In summary, ADSP stabilized the OS system through steric hindrance and electrostatic interaction.

### 3.5 FTIR

**Figure 2:**
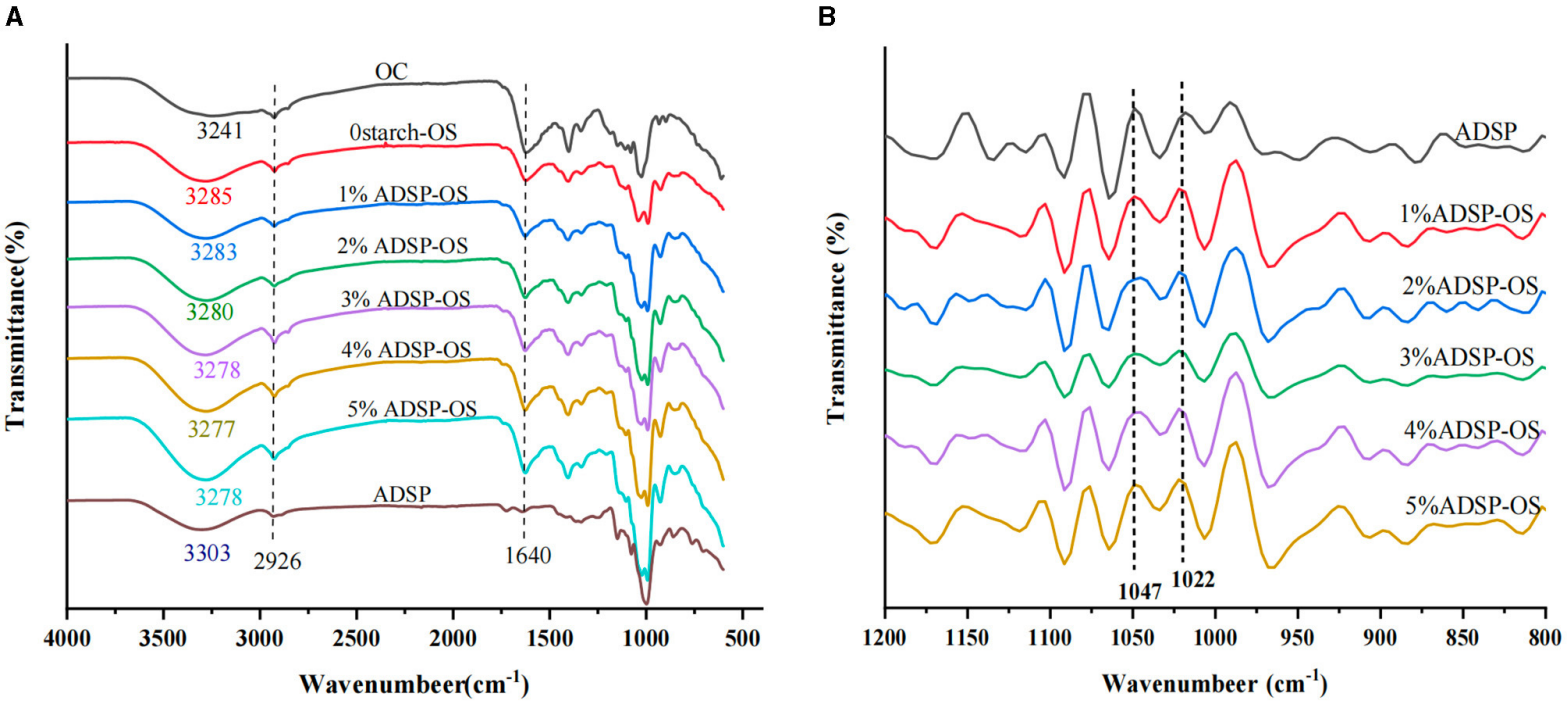
**(A)** FTIR spectra of OS, ADSP, and OS with or without starch; **(B)** 1,200–800 cm^−1^ infrared spectra after deconvolution.

The FTIR spectra of ADSP, OS, 0starch-OS, and ADSP-OS samples were investigated to further verify the interactions between starch and the OS system. As shown in Figure 2, the wide and large absorption bands between 3,100 and 3,600 cm^−1^ corresponded to the presence of O–H groups between polymers, which were formed by intramolecular hydrogen bonding (the same molecule) or intermolecular hydrogen bonding (adjacent molecules) (32). With an increase in the ADSP content, the hydroxyl stretching vibration peak shifted to a lower wavenumber (Figure 2A). According to the harmonic oscillator model, the enhancement of hydrogen bonding degree and strength could result in a decrease in the wavenumber (33). The results indicated that the addition of ADSP promoted the formation of stronger hydrogen bonds between starch molecules and the OS system. Moreover, the intensity of the peaks, ~3,300 cm^−1^, significantly increased, indicating an increased number of hydrogen bonds (33). No new peaks formed in ADSP-OS compared with 0starch-OS, suggesting that there was no covalent interaction between ADSP and the OS system. The absorption peaks in the region of 2,926 and 1,640 cm^−1^ were attributed to C–H stretching vibration and O–H bending vibration, respectively. The 1,640 cm^−1^ peak could represent the content of tightly bound water molecules in samples. The strength bonds of ~1,640 cm^−1^ in ADSP-OS corresponded to the higher tightly bound water content. These results were consistent with the WHC and LF-NMR measurements.

Moreover, the ratio of 1,047 and 1,022 cm^−1^ was calculated (Figure 2B) after deconvoluting the 1,200–800 cm^−1^ region to reflect the short-range ordering of starch. As shown in Figure 2B, Table 2, the addition of ADSP significantly reduced the 1,047/1,022 ratios of the OS (*P* < 0.05), further confirming that the OS system broke the hydrogen bonds between starch molecules, which was beneficial to the movement of starch chains, resulting in a strong hydrogen bonding between starch and the OS system. The small-molecule proteins might adhere to or enter the interior of starch, thus improving the molecular order, which manifested as an increase in the R1047/1022 values with increasing starch concentrations. These results indicated that not only electrostatic interactions and steric hindrance but also hydrogen bond interactions influenced the ADSP-OS system.

### 3.6 Rheological properties

**Figure 3:**
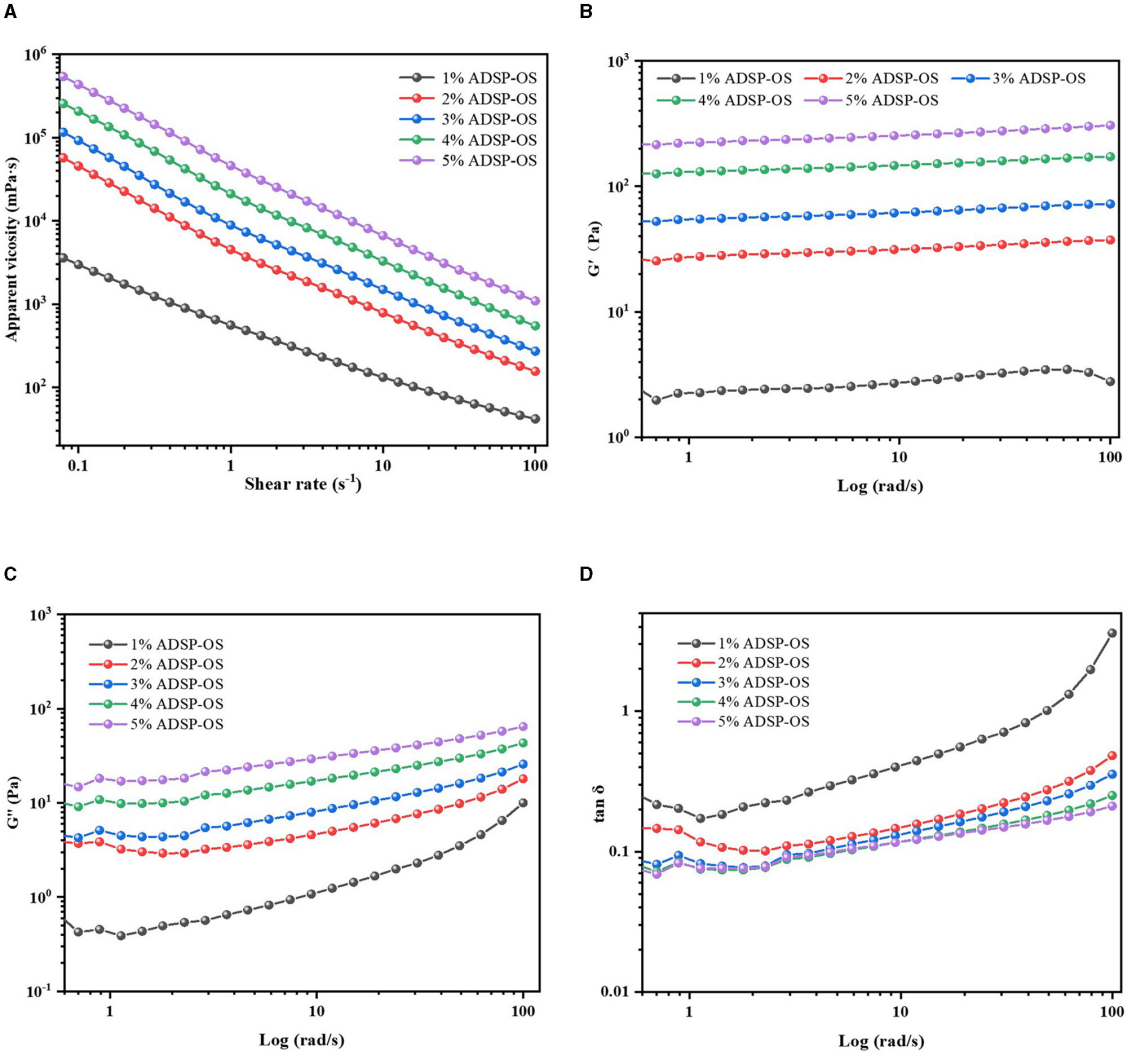
Steady shear rheological behavior of different OS **(A)**; dynamic frequency sweep curves **(B, C)**, and tan δ **(D)** of different OS.

The rheological properties of sauce are a crucial index of their application and processing properties. The flow properties of ADSP-OS are shown in Figure 3, Table 3. All samples demonstrated pseudoplastic fluid characteristics (shear thinning), characterized by decreasing viscosity in response to a rising shear rate, which was also confirmed by n values fitted using the power law model (*n* < 1). In the static state, the starch polymers were entangled with each other or other OS components, forming a stable structure. With the shear rate increasing, the intramolecular and intermolecular associations were broken, and the polymer chains started to disentangle, which led to disintegration in the structural OS and a reduction in the internal resistance of the sauces, which reduced the viscosity (34). The apparent viscosity increased with the increasing ADSP content. There are several reasons. First, as observed earlier, adding ADSP reduced the particle size of the OS, leading to an increase in the number of contacts and interactions between starch and starch or between starch and protein. In addition, due to dual-modification by acetic anhydride esterification and phosphate cross-linking, ADSP exhibited better water-binding capacity and showed excellent shear resistance (35). Therefore, increasing the ADSP concentration would increase the viscosity of the entire OS system. In addition, with the ADSP concentration increasing from 1% to 5%, the n value of the OS decreased from 0.47 to 0.19, but the K value increased from 0.50 to 44.01 Pa.sn. The results revealed that ADSP had a significant effect (*P* < 0.05) on the OS system, and the higher ADSP dosage could help enhance the pseudoplasticity of the OS. Overall, these results were consistent with the studies on other starch-based sauces (36–38).

**Table 3:** Power law parameters of different ADSP-OS.

**Sample**	**K**	***n***	**R^2^**
1% ADSP-OS	0.50 ± 0.01^e^	0.47 ± 0.02^a^	0.993
2% ADSP-OS	4.24 ± 0.05^d^	0.27 ± 0.01^b^	0.995
3% ADSP-OS	8.65 ± 0.03^c^	0.24 ± 0.00^c^	0.995
4% ADSP-OS	20.50 ± 0.18^b^	0.21 ± 0.01^d^	0.994
5% ADSP-OS	44.01 ± 0.44^a^	0.19 ± 0.01^d^	0.989

Different lowercase letters in the same column indicate significant differences (P < 0.05).

The storage modulus (G′), loss modulus (G″), and loss factor (tan δ = G″/G′) of ADSP-OS are shown in Figure 3. All OS had higher G′ values than G″ values during the studied frequency range, indicating the characteristics of solid-like behavior (typical of weak gels) (39). Moreover, with frequency change, all G′ values were almost parallel, exhibiting elasticity, which was dominant in the OS. The G′ and G″ of ADSP-OS enhanced with increasing ADSP concentration, suggesting an increase in the firmness and viscosity of the OS. This finding was consistent with the results of texture and static rheology. ADSP functioned not only as a filler but also as a supporting component, which is capable of increasing the G′ and enhancing the strength of the sample (40). Tan δ is an essential index for evaluating the viscoelastic behavior. The tan δ values of ADSP-OS decreased as the ADSP concentration increased (1%−5%, w/w), indicating that the addition of ADSP promoted the OS structure to convert from a more liquid-like form to a more solid-like form. A similar phenomenon was reported by Cui et al. (27). Due to the high dosage of ADSP, the degree of entanglement of swelling molecules could be strengthened, resulting in a strong structure. Increased shearing speed caused a significant increase in the values of tan δ due to the disintegration of starch granules and the disruption of OS architecture. Moreover, 1% ADSP-OS could not form a continuous structure.

### 3.7 Fluorescence micrographs

**Figure 4:**
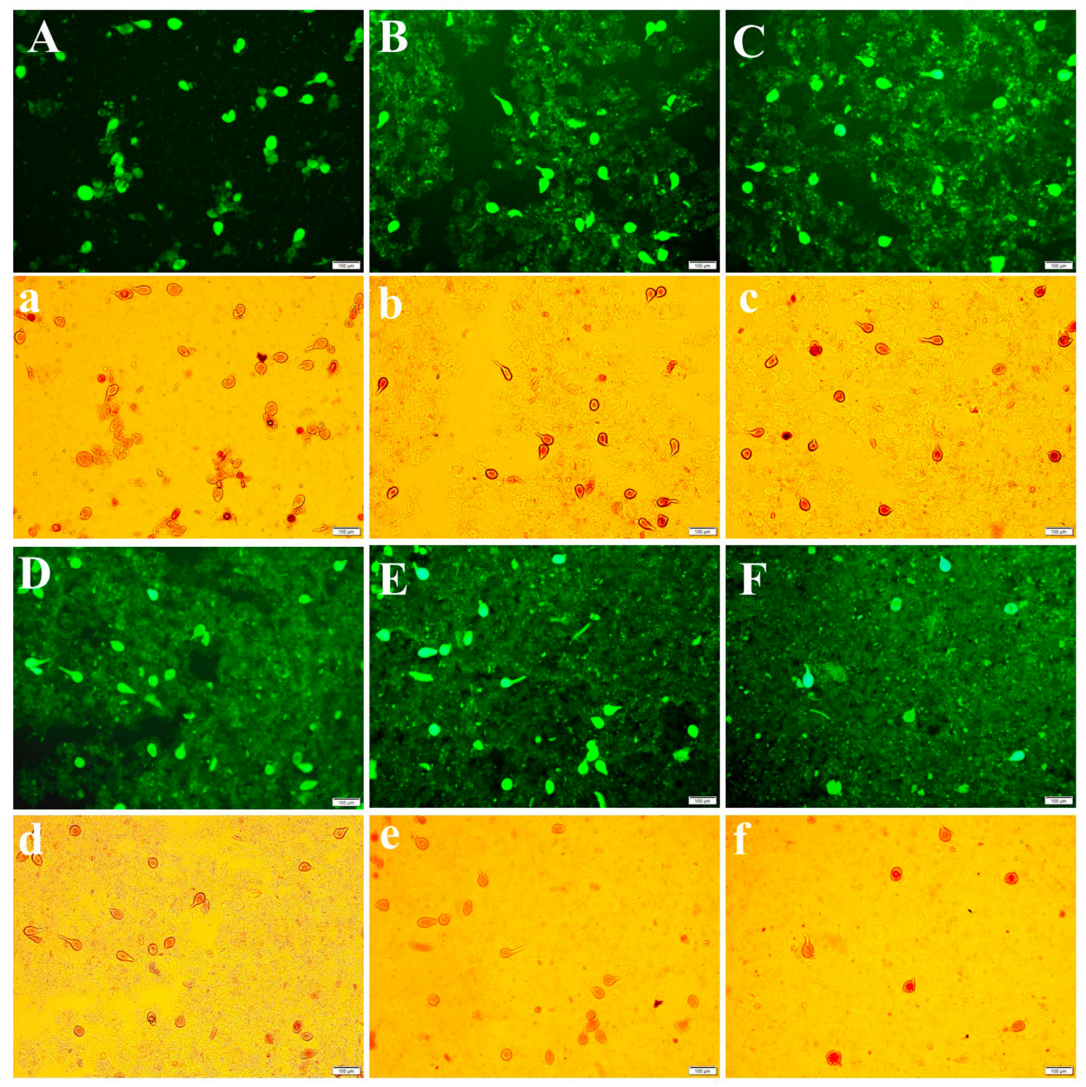
Fluorescence microscopy of OS: **(A, a)** 0starch-OS; **(B, b)** 1%ADSP-OS; **(C, c)** 2% ADSP-OS; **(D, d)** 3% ADSP-OS; **(E, e)** 4% ADSP-OS; and **(F, f)** 5% ADSP-OS.

The morphology of the OS with different ADSP proportions is shown in Figure 4. The protein appears in bright green, whereas the dimmer region represents starch. As seen in Figure 4A, a, the 0starch-OS system tended to aggregate and existed as large aggregates. Notably, the presence of ADSP weakened this aggregation. A looser structure with larger pores was observed in the OS system with 1%−2% ADSP. This phenomenon could be explained by the fact that the lower concentration of starch was insufficient to effectively disperse aggregates.

Moreover, the interstice of OS decreased significantly due to the filling effect of higher ADSP concentrations (3%−5%) (Figure 4). In addition, a larger number of ADSPs had a thickening and dispersing effect by increasing the concentration of the continuous phase. Moreover, a more uniform and denser microstructure was formed, which was beneficial in reducing the water mobility of the OS system. Interestingly, ADSP exhibited intact granules, which was helpful for the maintenance of structure. Similar results have been reported, indicating that ADSP is a rigid filler and that the complete particle is relevant for the characteristics of soybean protein gel (15).

### 3.8 AFM

**Figure 5:**
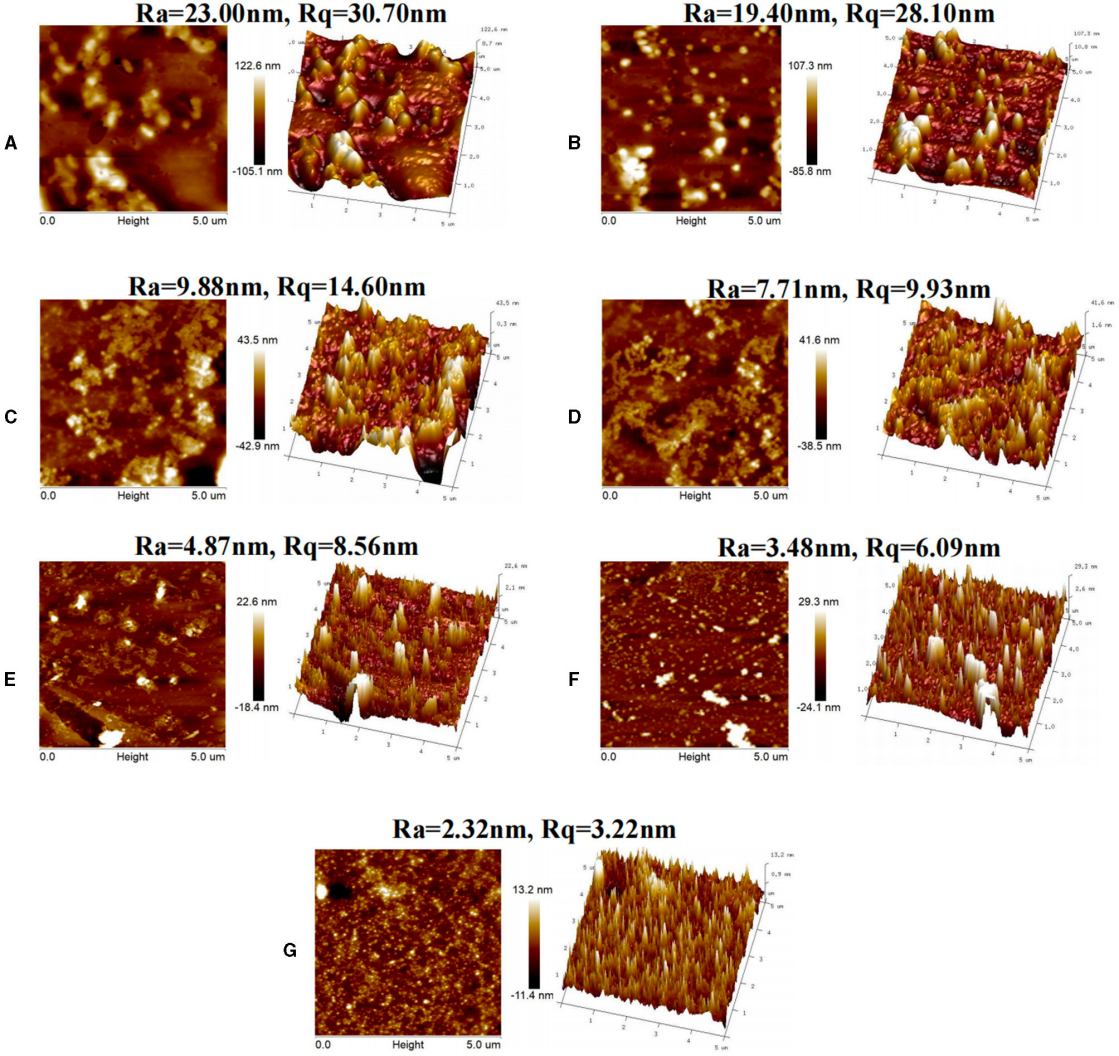
AFM images of 0starch-OS **(A)**; 1%ADSP-OS **(B)**; 2%ADSP-OS **(C)**; 3%ADSP-OS **(D)**; 4%ADSP-OS **(E)**; 5%ADSP-OS **(F)**; and ADSP **(G)**, respectively.

AFM is a high-resolution microscopy technique that can reflect the surface topography of samples at the nanoscale using tiny force-sensitive devices (41). In the current study, the morphology of the OS system was determined using AFM to observe the aggregation shape. As shown in Figure 5, the deposition of 0starch-OS on mica sheets appeared as a stack of large components. It had the highest roughness parameters: an average surface roughness (Ra) value of 23.00 nm and a root mean square roughness (Rq) value of 30.70 nm, confirming the accumulation of OS protein in the 0starch-OS system. The Ra values of 1% ADSP-OS, 2% ADSP-OS, 3% ADSP-OS, 4% ADSP-OS, and 5% ADSP-OS were 19.40, 9.88, 7.71, 4.87, and 3.48 nm, respectively. Moreover, the starch granule surface was smooth (Ra 2.32 nm, Rq 3.22 nm). As expected, the ADSP addition reduced the height of the OS structure, which could be attributed to ADSP limiting the movement and aggregation of proteins by increasing the viscosity of the OS system. Moreover, ADSP filled in the OS system and created stronger steric hindrance, thereby inhibiting the migration of proteins. In contrast, low dosages of ADSP (1%−2%) showed a poor filling effect compared with the other concentrations. These results were in line with fluorescence microscope observations.

### 3.9 Physical stability

**Figure 6:**
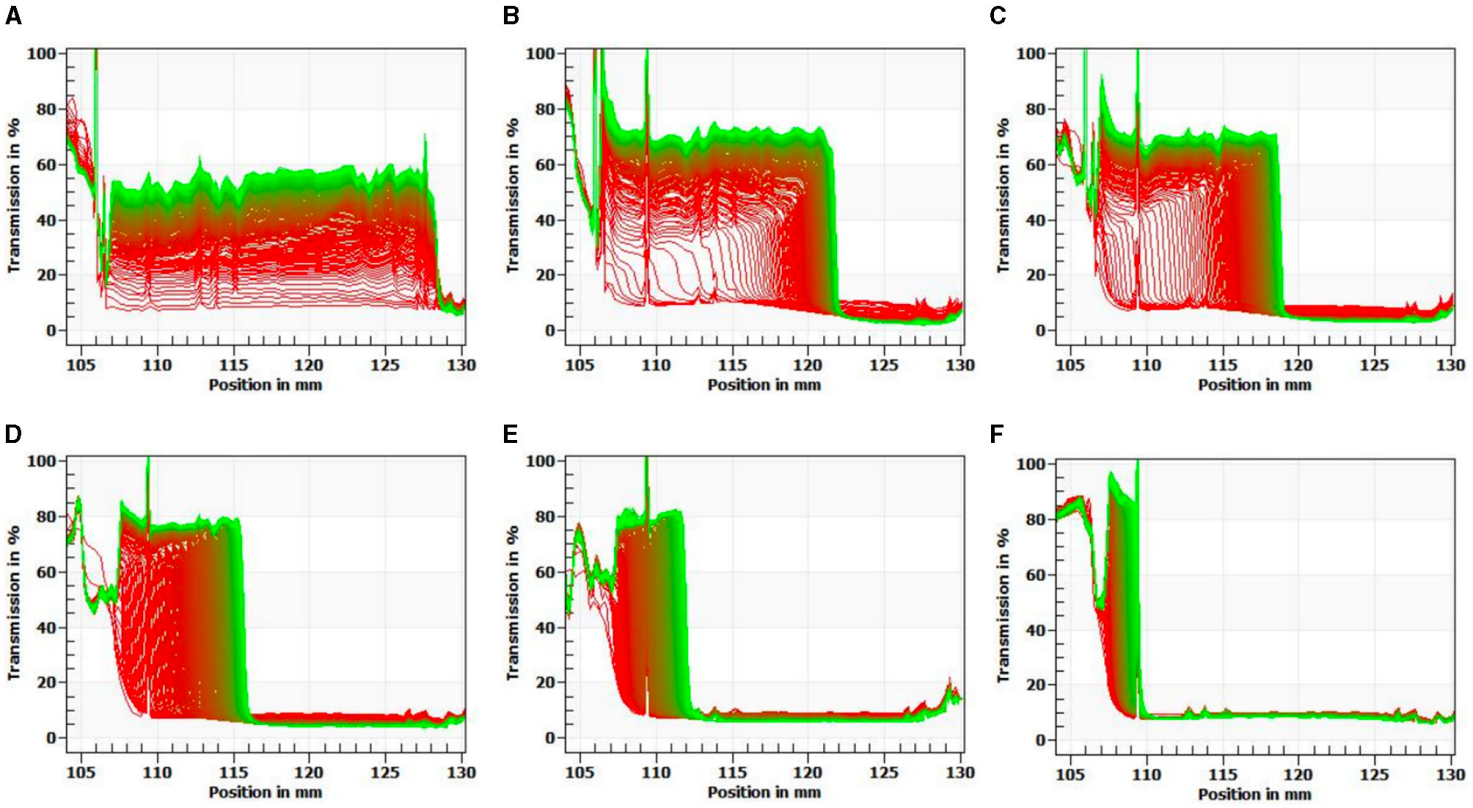
Near-infrared transmission extinction profiles of 0starch-OS **(A)**; 1%ADSP-OS **(B)**; 2%ADSP-OS **(C)**; 3%ADSP-OS **(D)**; 4%ADSP-OS **(E)**; and 5%ADSP-OS **(F)**, respectively.

**Figure 7:**
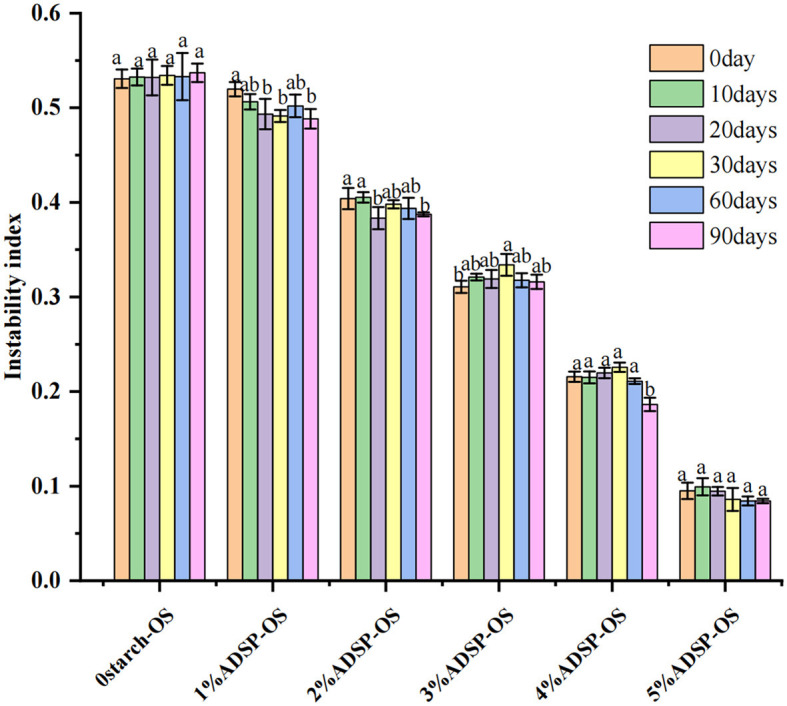
Instability index of different OS after 0, 10, 20, 30, 60, and 90 days of storage. Different letters indicate significant differences (*P* < 0.05).

Centrifugation stability is an important manifestation for evaluating sauce stability. The physical stability of the OS was measured using LUMiFuge. This instrument recorded the Near Infrared (NIR) light transmittance at different locations to reflect the migration process of particles. Moreover, the slope of the transmission extinction profile was defined as an instability index by the data treatment (42). Figure 6 exhibits the message on sedimentation fractions of the OS system stabilized by ADSP with different concentrations. The abscissa indicates the sample position regarding the bottom of the sample tube, and the ordinates are related to the light transmittance of the sample. The transmission profiles can reveal the movement of the interface by comparing the light transmittance changes. In the current study, the high transmission on the left represents fluids, whereas the low transmission on the right relates to sediment. The results indicated that the transmission profiles of ADSP-OS became lower with increasing ADSP content, suggesting that the stability of the OS was enhanced. In 5% ADSP-OS, almost no layer appeared. These results are in line with the instability indexes (Figure 7). A higher instability index represents lower sample stability. Higher ADSP concentrations contributed to the retention of water. However, regardless of the ADSP concentration being high or low, all samples exhibited a similar instability index during long-term storage, suggesting the excellent stability of ADSP in OS systems. Additionally, the transmittance of ADSP-OS was higher than that of 0starch-OS, which suggested that ADSP improved the transparency of the OS.

## 4 Conclusion

In the present study, it was observed that ADSP enhanced the stability of the OS. Adding ADSP provided a paste-like texture for OS and increased the firmness, consistency, WHC, G′, G″, water retention capacity, and bound water content of the OS system. As a fence-type long-chain molecule, ADSP hindered the contact and aggregation of proteins in the OS, which manifested as a reduction in particle size. Compared with 0starch-OS, hydrogen bonding was also strong in the ADSP-OS system. In addition, ADSP could maintain the integrity of its granules under extreme conditions (low pH and high temperature) and provide a good filling effect for oyster oil systems. Therefore, adding an appropriate amount of ADSP could improve the stability of the OS and increase its shelf life.

## Data availability statement

The original contributions presented in the study are included in the article/supplementary material, further inquiries can be directed to the corresponding authors.

## Author contributions

XL: Formal analysis, Methodology, Software, Writing – original draft. FZ: Investigation, Writing – review & editing. XK: Data curation, Investigation, Writing – review & editing. WG: Data curation, Investigation, Writing – review & editing. BC: Conceptualization, Resources, Supervision, Writing – review & editing. JS: Conceptualization, Resources, Supervision, Writing – review & editing.
